# Surveillance of COVID-19 outbreaks in prisons in the US South: The role of economic distress in the communities surrounding prison facilities

**DOI:** 10.1017/cts.2022.432

**Published:** 2022-08-01

**Authors:** Mofan Gu, George Pro, Nickolas Zaller

**Affiliations:** 1 Department of Health Behavior and Health Education, University of Arkansas for Medical Sciences, Fay W. Boozman College of Public Health, Little Rock, AR, USA; 2 Southern Public Health and Criminal Justice Research Center, University of Arkansas for Medical Sciences, Fay W. Boozman College of Public Health, Little Rock, AR, USA

**Keywords:** Health disparities, prison, economic stability, social determinant of health, COVID-19

## Abstract

**Introduction:**

The US South is the epicenter of the epidemic of mass incarceration. Prisons have experienced substantial challenges in preventing COVID-19. Incarcerated individuals and prison staff are at a high risk for infection due to minimal available preventive measures. Prisons are not closed systems and many staff come from communities in close proximity to the facility. Characteristics of the communities immediately surrounding prisons are an overlooked but critical factor to better understand the role prisons play in pandemics.

**Methods:**

We used facility-level COVID-19 data from the COVID Prison Project to identify the number of unique outbreaks between May 2019 and May 2020. We used a county-level composite indicator of economic distress (DCI score) to identify the environment surrounding each prison (2015–2019). We modeled the number of outbreaks to DCI scores using negative binomial regression, adjusting for race/ethnicity (African American and Latino/Hispanic), age (65 and older), and rurality level.

**Results:**

Our sample included 570 prisons in 368 counties across 13 Southern states. We found that score was positively and significantly associated with prison COVID-19 outbreaks (aRR, 1.012; *p* < 0.0001), and rurality was potentially a stronger surrogate measure of economic distress (aRR, 1.35; p, 0.02). Economic stability is a key precursor to physical health. Poorer communities have been disproportionately impacted by the pandemic, and we found that prisons located in these communities were more susceptible to recurring outbreaks. Prison-based disease prevention interventions should consider the impact that the outside world has on the health of incarcerated individuals.

## Introduction

Coronavirus disease (COVID-19), caused by the SARS-CoV-2 virus, has led to a global pandemic and impacted millions of Americans, especially vulnerable populations like incarcerated individuals [1–4]. Between the beginning of the pandemic (March 2020) and August 2020, ninety of the largest 100 COVID-19 cluster outbreaks in the USA occurred in prisons and jails [5]. Nationally, as of 8 Jun 2022, there have been 588,711 cases and 2,894 deaths have been among incarcerated individuals [5]. As of November 2021, the COVID-19 incidence rate in carceral settings was 3.7 times higher than the national rate; in addition, incarcerated individuals are two to three times more likely to die from COVID-19 [6–8]. Case fatality rates among incarcerated individuals vary by state, with the highest rate observed in Alabama (24 deaths per 1000 cases) [5]. As new variants emerged into 2022, the incidence and mortality rates continued to be disproportionately higher in correctional facilities [8].

Correctional settings are prone to COVID-19 outbreaks for several reasons. First, incarcerated populations are more vulnerable to exposure, infection, and serious adverse outcomes, due to high prevalence of chronic diseases (e.g., asthma, cancer, hepatitis C, and HIV) and mental health disorders [9,10]. With harsh sentencing policies, incarcerated populations are aging and suffer from geriatric conditions that make them even more vulnerable [9,11]. Between 1999 and 2016, the number of individuals in US prisons aged 55 years and older grew 280%, as compared to 3% growth in younger age groups [11]. Second, correctional facilities serve as disease incubators. Prisons and jails frequently suffer from overcrowding, poor ventilation, unsanitary living conditions, limited access to health care, and understaffing, all increasing the risk of COVID-19 transmission [9,10,12]. Many of these risk factors are disproportionately more prevalent in the South [13]. The lack of correctional staff, especially ones appropriately trained to provide healthcare, adds additional burden to a facility’s ability to quickly and effectively prevent and/or control COVID-19 outbreaks [12]. Finally, correctional staff may aid COVID-19 transmission through their close and prolonged contact with incarcerated individuals. Similar to incarcerated individuals, correctional staff experienced higher COVID-19 incidence and mortality than the general public. As of June 2022, there have been 204,839 confirmed COVID-19 cases and 278 deaths among prison staff in the USA [5]. COVID-19 outbreaks in correctional facilities potentially influence the counties they reside in, as hundreds of thousands of correctional staff, vendors, and visitors travel frequently between communities and prisons [12,14]. In addition, incarcerated individuals may also be released or transferred to other facilities [12,14].

System-level factors, such as rurality, economic stability, and racial disparity, likely contribute to a county’s vulnerability to prison-community COVID-19 transmission. Since summer of 2020, higher incidence rates and mortality rates have been observed in rural America [15]. Rural communities in the USA usually experience health disparities, due to unique geographic (e.g., distance to hospitals), socioeconomic (e.g., food and housing security), and other factors (e.g., racism and discrimination) [16]. Economic stability is a social determinant of health which encompasses employment, income, housing security, and food security [17,18]. The link between economic instability and disease transmission is well documented in epidemiologic studies [19–25]. Racial disparity is a historical problem that is inextricably associated with socioeconomic status [26]. Throughout the pandemic, people of color in the USA had significantly higher rates of infection, hospitalization, and mortality as compared to their White counterparts [27,28]. These populations may have jobs that could not be performed remotely, live/work in crowded conditions, suffer from pre-existing conditions that makes them even more vulnerable to COVID-19, and lack access to good nutrition and health care [26].

Economic crisis has long-term impacts on infectious disease transmission and control, and prison populations are especially vulnerable to epidemics during economic duress [29]. A recent study found a significant association between county socioeconomic status and community COVID-19 transmission [30]. COVID-19 spread in prisons could potentially be fueled by community transmissions in the county, which in turn are affected by economic stability and other social determinants of health. However, published studies rarely examined the association between a county’s economic well-being and prison COVID-19 transmission. Controlling the spread of COVID-19 in prisons is essential for preventing large outbreaks within surrounding communities. Better understanding the association between local socioeconomic status and COVID-19 transmission inside prisons could inform resource allocation during crises and future pandemics, while also more effectively controlling prison outbreaks and minimizing community transmissions.

In this exploratory study, we aim to assess the association between county economic distress and prison COVID-19 outbreaks in southern US states, where COVID-19 and mass incarceration are prevalent. Findings may contribute to an epidemiologic profile of how socioeconomic characteristics impact COVID-19 transmissions in and out of correctional systems and help understand the underlying mechanisms to better combat the COVID-19 pandemic as well as future pandemics.

## Materials and Methods

### Outcome Definition

The outcome of interest for the current study was the number of COVID-19 outbreaks in prisons within a county, between May 2019 and May 2020. In our study, a prison COVID-19 outbreak was defined as when the daily incidence was one standard deviation above the total mean prison incidence rate during the study period. Observations with values above one standard deviation from the mean are typically considered abnormally high [31]. Several epidemiologic studies have identified disease outbreaks using this definition [32,33]. The original data set contained daily COVID-19 cases in each prison. We first geocoded each prison in ArcGIS Pro 2.6 (ESRI, Redlands CA) using the facility name and address, then aggregated total COVID-19 cases in prisons for each county using the coordinates generated in the maps. Since some counties had more than one prison, daily incidence rate was calculated for each prison first, and then averaged at the county-level to identify COVID-19 outbreaks. We then counted the number of outbreaks within the study period for each county.

We obtained prison COVID-19 data from the COVID Prison Project (CPP), which aggregates data daily on COVID-19 incidence in incarcerated individuals and correctional staff in US states and territories [5]. Data were extracted and aggregated from 53 prison systems and media reports daily, using both manual and automated approaches [5]. We included 570 prisons in 368 counties across 13 Southern US states in the study: Alabama, Florida, Georgia, Kentucky, Louisiana, Missouri, Mississippi, North Carolina, South Carolina, Tennessee, Texas, Virginia, and West Virginia.

### Exposure and Covariates

We used the Distressed Community Index (DCI), a composite score of economic distress, as a measure of a county’s economic distress [34]. The DCI score is set on a scale of 0 to 100, with 100 being the most distressed. It is derived from U.S. Census Bureau’s American Community Survey 5-Year Estimates (2015–2019), using seven individual subscales: education (percentage of people aged 25 and older without a high school diploma or equivalent), housing vacancy (percentage of habitable, unoccupied housing), unemployment (percentage of unemployed individuals aged between 25 and 54 years), poverty (percentage of population living under the federal poverty line), income (median house income over metro area household income), job stability (percent change in number of jobs), and establishment (percent change in number of business establishments) [34]. In addition to DCI score, we also assessed several county-level sociodemographic variables as potential confounders: racial composition (percentages of African Americans and Hispanic/Latino Americans), elderly population scale (percentage of population aged 65 and older), and rurality level of the county. Data on race and age were sourced from the American Community Survey (2015–2019), and rurality was based on the CDC’s Rural-Urban Classification (2013), where US counties were scored on a scale of one to six (six being the most rural) [35].

### Statistical Analysis

We first compared prison COVID-19 outbreaks and socio-demographic measures (DCI score, percentage of African Americans, percentage of Latino/Hispanics, and percentage of people aged 65 years and older) at the state-level to assess any differences by state. We used t-tests to compare individual DCI measures between counties, stratified by whether a prison outbreak had occurred. We also created a bivariate choropleth map in ArcGIS to better visualize COVID-19 outbreaks and DCI score geographically.

For regression modeling, we first created bivariate negative binomial regression models between the number of prison COVID-19 outbreaks and the DCI score to assess crude association, using the natural log of the county’s total population as offset. Then we created two multivariate regression models, one with the DCI score, race (percentage of African American and percentage of Latino/Hispanic), and age (percentage of aged 65 and above) and the other with DCI score, race, age, and rurality level, to assess adjusted associations. All statistical analyses were performed in SAS 9.4 (SAS Institute, Cary, NC).

## Results

We included 570 prisons in 368 counties across 13 Southern US states in our analyses: Alabama, Florida, Georgia, Kentucky, Louisiana, Missouri, Mississippi, North Carolina, South Carolina, Tennessee, Texas, Virginia, and West Virginia. Characteristics of each state were summarized in Table 1. South Carolina had the lowest mean Score (51.7), and Louisiana had the highest (80.2). At the county level, Stafford County in Virginia had the lowest DCI score (1.3), and McDowell County in West Virginia had the highest (100). One hundred and forty-six counties had no prison COVID-19 outbreaks using our definition, and Fluvanna County in Virginia had the largest number of prison COVID-19 outbreaks (37).

**Table 1.** Number of outbreaks and socioeconomic status by state*

State	Distress Community Index (DCI) Score^‡^			Average Number of Outbreaks^*†*^	Percent African American^‡^	Percent Hispanic/Latino^‡^	Percent above 65^‡^	Number of Facilities^*†*^
	Mean	Min	Max					
Alabama	60.6	20.1	94.8	1.53	31.0%	3.6%	16.7%	32
Florida	55.2	8.8	97.0	3.33	15.6%	12.6%	23.1%	60
Georgia	66.9	4.0	99.6	0.89	33.0%	6.5%	13.5%	140
Kentucky	70.5	5.5	99.9	4.30	4.4%	2.2%	12.8%	14
Louisiana	80.2	54.0	99.4	2.00	37.6%	2.5%	17.5%	10
Missouri	78.3	12.6	99.6	0.87	49.1%	2.7%	15.6%	23
Mississippi	62.0	21.9	95.2	2.61	6.3%	2.9%	15.9%	22
North Carolina	55.3	1.9	95.9	4.11	22.1%	8.0%	17.6%	62
South Carolina	51.7	7.2	95.0	2.19	39.4%	5.2%	16.4%	21
Tennessee	70.5	15.9	92.7	2.18	19.9%	3.2%	12.5%	15
Texas	68.3	2.5	99.5	2.84	9.9%	34.2%	14.0%	112
Virginia	64.2	1.3	99.7	3.97	21.6%	3.9%	18.9%	38
West Virginia	69.1	10.6	100.0	1.89	3.2%	1.3%	19.4%	21

*Based on counties selected in the study.

†Number of outbreaks and number of facilities based on data from May 2019 to May 2020.

‡Demographic information based on American Community Survey (2015-2019 5-year average).

When comparing counties that had prison-based COVID-19 outbreaks during the study period with counties that did not, housing vacancy and unemployment stood out as significant risk factors (Table 2). Prison COVID-19 outbreaks were significantly more likely to occur in counties with high housing vacancy rates and high unemployment rates. One counterintuitive finding was that COVID-19 outbreaks in prisons were inversely associated with poverty rates.

**Table 2.** Distressed Community Index (DCI) metrics in counties with and without prison COVID-19 outbreaks

	Prison COVID-19 Outbreaks		t-test
	Yes	No	p
No High School Diploma	13%	12%	0.20
Housing Vacancy*	15%	13%	0.02
Adult Not Working*	32%	31%	0.01
Poverty*	17%	20%	0.0006
Median Income Ratio	86%	84%	0.35
Increase in Employment	3%	5%	0.12
Increase in Establishment	3%	1%	0.07

*Statistically significant, based on alpha value of 0.05.

Results from bivariate and multivariate models are shown in Table 3. In the bivariate model, DCI score was significantly associated with the number of prison COVID-19 outbreaks based on an alpha value of 0.05. Higher DCI score was associated with more prison outbreaks in a county—with one unit increase in DCI score, the number of outbreaks increases by 1.02 times. The composite score of DCI remained positively and significantly associated with the number of prison COVID-19 outbreaks, when adjusting for racial composition and elderly population scale of the county. However, when we added rurality to the multivariate model, higher rurality, instead of higher DCI score, was found to be associated with more prison outbreaks.

**Table 3.** Regression analysis between socioeconomic measures and prison COVID-19 outbreaks

	Rate Ratio	95% Confidence Interval	p
**Bivariate Model**			
DCI score*	1.02	(1.01, 1.02)	<0.0001
**Multivariate Model 1**			
DCI Score*	1.02	(1.01, 1.03)	<0.0001
% African American*	0.047	(0.007, 0.31)	0.001
% Hispanic/Latino*	0.057	(0.007, 0.47)	0.008
% Aged 65 and above	1.44	(0.0075, 279.14)	0.89
**Multivariate Model 2**			
DCI Score	1.01	(1, 1.02)	0.13
% African American*	0.069	(0.011, 0.42)	0.004
% Hispanic/Latino*	0.11	(0.013, 0.94)	0.04
% Aged 65 and above	0.19	(0.0013, 28.16)	0.52
Rurality*	1.35	(1.06, 1.73)	0.02

*Statistically significant, based on alpha value of 0.05. DCI, Distressed Community Index.

**Fig. 1.**
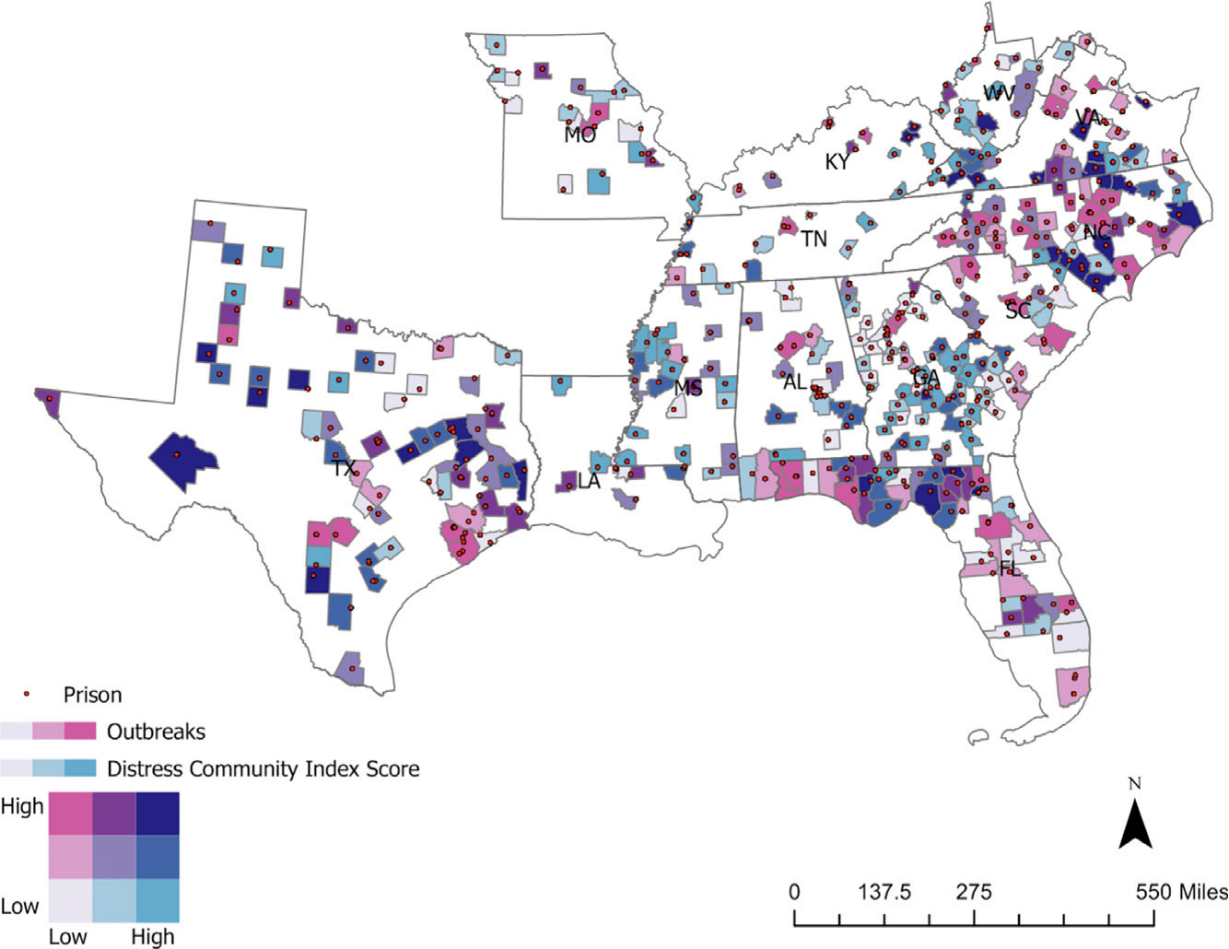
Prison locations, distribution of COVID-19 prison outbreaks, and community economic distress in the US South, May 2019 - May 2020.

We created a bivariate choropleth map to visualize prison COVID-19 outbreaks and DCI score (Fig. 1). The geographic distribution of prison outbreak severity and DCI score varied across the 13 states. Of note, there were 46 prisons in 33 counties that had both high numbers of outbreaks and high distress scores (shown as dark purple in the map).

## Discussion

This study demonstrates how economic distress in US counties impacts COVID-19 outbreaks in prisons. Social and structural determinants of health disproportionately affect incarcerated populations, making them more vulnerable to rapid transmission of COVID-19 [36]. Based on our multivariate regression models, the composite DCI score was significantly associated with the number of prison COVID-19 outbreaks within the county. Distressed communities with lower socioeconomic status experience substantially higher risk of prison COVID-19 outbreaks. Our findings align with previous studies that identified economic distress as a risk factor for community COVID-19 transmission [19–24,30]. Community outbreaks and prison outbreaks closely influence one another, as studies found that staff, vendors, and visitors serve as a vehicle for disease transmission between prisons and communities [12,14]. Economically distressed communities potentially have higher COVID-19 prevalence, increasing the risk of introducing the virus to prisons and starting outbreaks. Moreover, the community-prison transmission is potentially complex and bidirectional and could form a vicious cycle in distressed communities with inadequate resources and healthcare infrastructure. Incarceration is known to be negatively associated with community health [29,37]. Several empirical studies have found that the presence of prisons, as well as their resident capacity, were significantly associated with COVID-19 rates within the county [12,38]. Such association persisted even with implementation of systemwide mitigation efforts (e.g., hygienic precautions, isolation, and quarantine) [12].

Among the individual subscales, unemployment was found to significantly increase the risk of prison COVID-19 outbreaks. Understaffing potentially makes prisons more vulnerable to rapid dissemination of infectious diseases [12]. High unemployment rates of the community might indicate understaffing in prison and more challenges in preventing and controlling COVID-19 outbreaks. In addition, unemployment potentially drives up crime rates in the county and may consequentially lead to higher incarceration rates and higher risks of prison outbreaks due to prison overcrowding [39,40]. Future research may consider how factors related to economic health and employment prospects affect staffing shortages in prisons.

One interesting finding is that rurality appeared to be more strongly associated with prison COVID-19 outbreaks than DCI score. When both rurality and DCI score were in the multivariate model, only rurality remained statistically significant. In further subanalyses, rurality and DCI were significantly correlated (*r*, 0.70; *p* < 0.0001). This indicates that the rurality scale could potentially be a better proxy measure of county economic health. Rurality and economic distress have both been found to increase risk of COVID-19 transmission [15,19–24,30]. They are known to be closely associated with one another—rural communities in the USA commonly face economic disparities (e.g., poverty and poorer social infrastructure) [41]. In our study, a county’s rurality was highly correlated with its DCI score (*r* = 0.7, *p* < 0.0001). As we know, area-level socioeconomic status could not be measured directly with traditional measures like income [41]. Vlahov and Galea discussed in a commentary how rurality was commonly used as a surrogate for social determinant of health in epidemiologic studies, as rurality/urbanicity tends to capture aspects of physical environment, social environment, and access to health and social services [42]. Therefore, it makes sense that both DCI score and rurality were positively associated with prison COVID-19 outbreaks; our study indicates that rurality potentially did a better job to capture economic disparities and to explain prison outbreaks. Considering how many prisons are located in rural towns in the USA, public health practitioners need to pay extra attention to rural America when it comes to COVID-19 prevention and mitigation [43]. In addition to resource allocation, we also need to work on health education, infrastructure building, and engaging rural residents, so people are aware that prison and community COVID-19 transmission are not exclusive to urban areas.

A counterintuitive finding in our study was the inverse association between poverty and prison outbreaks. Interestingly, Hawkins et al. observed a similar association between poverty and community COVID-19 outbreaks, using the same county-level poverty data from American Community Survey [30]. More future studies are needed to explain this finding—at the moment, we could not know whether it is due to some unknown mechanism, data not adequately capturing “true” poverty status, residual/unknown confounders, or other reasons.

Our study informs community preparedness and mitigation efforts for COVID-19. First, our study emphasized the urgent need to understand the dynamic of prison-community COVID-19 transmission. Correctional health is community health. Prisons were the hardest and fastest hit during the COVID-19 pandemic, and transmission between prisons and communities work in both directions. Socioeconomic disparities of the community make it more challenging to stop such transmissions. Therefore, addressing social determinant of health is crucial in COVID-19 preparedness and mitigation. When a community is economically stable, it would have better healthcare infrastructure, social support network, and more adequate staffing and COVID-19 protocols in prisons, making it more resilient to disease outbreaks and other adverse events. Second, our study could help identify communities most vulnerable to prison COVID-19 outbreaks. We found rurality, unemployment, and housing vacancy rate as significant risk factors of prison COVID-19 outbreaks in the USA. We could potentially use these data to detect communities at highest risk of prison COVID-19 outbreaks in the future and better prepare them for disease prevention and control. In addition to proper protocols and adequate medical/sanitation supplies, our findings may also guide future funding to improve infrastructures in these high-risk communities and reduce community vulnerability. Some examples include supporting economic development in rural counties through investments in small local business and increased collaborations between health care and development sectors, considering high-risk communities as pilot sites for new programs/interventions, and creating sustainable programs that continue to learn about the local community and to identify opportunities to improve health [16]. Third, our study informs use of alternatives (probation, parole, and treatment programs) to reduce mass incarceration, depopulate overcrowded prisons, and potentially prevent outbreaks. We observed almost no early releases during the pandemic, despite discussions among lawmakers in the early phase. Changes in prison policies and legislations should be discussed and implemented in preparation of future outbreaks. Lastly, our study informs future research. CTSAs are uniquely positioned to leverage their existing resources to study how the health of incarcerated populations can impact community health. Future studies could better explore the mechanisms with more and better data.

Our study has limitations. The biggest limitation is data quality on COVID-19 incidence in prisons. There were potential errors in collection, reporting, and automated scraping. First, case counts might not represent the true incidence in prison due to lack of testing and changes in case definition over time. Second, we observed many zeros in daily cases; however, we could not know whether the zeros meant no new cases or lack of reporting. One possibility is that prisons reported cases on certain days of the week/month, resulting in large spikes in case counts which does no't necessarily indicate outbreaks. Third, the COVID Prison Project utilized automated data scraping methods and could lead to errors when data are presented on webpages in ways unrecognizable to the algorithm. To overcome some of these limitations, we performed rigorous data cleaning and checking and used the number of outbreaks as our outcome, instead of keeping the daily rates. Such outcome definition has its own limitations. We would not have been able to capture any outbreaks if the incidence rate remained high during the entire study period, since our definition of outbreak was a relative measure. This exploratory study serves as a preliminary assessment of economic distress and prison COVID-19 transmission. We found county economic distress, as well as rurality, to be positively associated with prison COVID-19. However, the underlying mechanism is complex and requires more studies. In future studies, we could include all 50 states in the USA and incorporate more time-dependent socioeconomic and behavioral data at the county level, as well as facility-specific characteristics (e.g., security and staffing level, overcrowding, and treatment availability), to help account for variation over time and residual confounding.
